# Necrotizing Soft Tissue Infection: A Single-Center Retrospective Study of Treatment and Outcomes

**DOI:** 10.7759/cureus.15039

**Published:** 2021-05-15

**Authors:** Dzemail Detanac, Mehmed Mujdragic, Dzenana A Detanac, Enes Zogic, Lejla Ceranic, Kemal Alihodzic, Mersudin Mulic, Hana Mujdragic

**Affiliations:** 1 Department of Surgery, General Hospital Novi Pazar, Novi Pazar, SRB; 2 Department of Ophthalmology, General Hospital Novi Pazar, Novi Pazar, SRB; 3 Department of Infectious Diseases, General Hospital Novi Pazar, Novi Pazar, SRB; 4 Department of Biomedical Science, State University of Novi Pazar, Novi Pazar, SRB; 5 Department of Anaesthesiology, General Hospital Novi Pazar, Novi Pazar, SRB

**Keywords:** general surgery, soft tissue infection, necrotizing fasciitis, fasciitis

## Abstract

Introduction

Necrotizing fasciitis is a severe inflammatory disease of the body's soft tissue characterized by spreading rapidly and high mortality. Rapid surgical intervention along with other supportive measures of treatment have a great impact on the outcome of treatment.

Material and methods

This study was conducted by a retrospective medical record review of all patients with a microbiologically and clinically confirmed diagnosis of necrotizing fasciitis who were admitted to the general surgery department at the General Hospital Novi Pazar, Serbia, during the period between 2017 and 2020. Demographic, clinical, laboratory, and microbiology data were analyzed.

Results

A total of 13 cases were identified, which represents 0.21% of the total number of patients treated at the surgical department during the period January 2017 to November 2020. The mean age of patients was 55 years, with a male/female ratio of 1:1.6. All of them had at least one comorbidity and more than half had three or more. Diabetes, cardiovascular diseases, and obesity were the most common comorbidities. The most common causes of infection were *Klebsiella spp, Pseudomonas aeruginosa, S. pyogenes, *and* S. aureus*. All patients received multiple surgical interventions (mean 2.3).

Conclusion

Treating necrotizing fasciitis requires a multidisciplinary approach. Early diagnosis and rapid clinical response allow for better disease outcomes. Getting to know more about necrotizing fasciitis will help doctors make better decisions when treating it.

## Introduction

Necrotizing fasciitis (NF) infections are rare and severe soft tissue infections characterized by rapid, progressive spreading among deep and superficial fascia and subcutaneous tissue, which can lead to shock and sepsis with multiorgan failure and a potentially fatal outcome [1].

Initial symptoms are tissue swelling, erythema, crepitations, fever, tenderness, odor, pain, bullous changes, and skin necrosis [2-3]. At first, the patient may be presented with cellulitis because of the similarity with NF, which then rapidly progresses with pain disproportionate to the area of infection. Early diagnosis failure and delay in appropriate treatment often result in a severe clinical picture, mutilating surgical procedures, and high mortality [4].

The incidence of this infection is 1-4/100,000 persons per year [3]. According to data from the literature, nearly 7-14,000 cases of all types of NF occur in the United States each year [5] and 4.8 deaths per 1,000,000 person-years [6]. The infection most commonly affects the anterior abdominal wall, perineum, and scrotum, as well as the extremities [3].

This study aims to present the frequency of NF treated in our hospital for better future monitoring and development of NF awareness in primary care physicians so that these patients can be referred to a higher medical level in time for appropriate treatment.

## Materials and methods

This study was conducted by a retrospective medical record review of all patients with a microbiologically and clinically confirmed diagnosis of necrotizing fasciitis, who were admitted to the department of general surgery, General Hospital Novi Pazar, Serbia, during the period between 2017 and 2020. The study was approved by the Ethics Committee of General Hospital Novi Pazar (798/2021), and it was conducted under the principles of the Declaration of Helsinki.

Laboratory and microbiology test results, patients' characteristics (sex, age, comorbidities, infected site), preoperative, postoperative, and surgical treatment, duration of hospitalization, and clinical outcome were analyzed.

## Results

During the period 2017-2020, 6067 patients were hospitalized at the department of surgery at General Hospital Novi Pazar, and out of these, 13 (0.21%) patients were treated with NF. There were five (38.5%) male and eight (61.5%) female patients treated with NF, with a mean age of 55 years (range 27-73 years) and an approximate M/F ratio of 1:1.6.

Seven patients were admitted to the department of surgery through the emergency surgical clinic referred by general practitioners, two were transferred from a tertiary health institution after surgery (both due to gynecological malignant tumors), and four patients were transferred from the endocrinology department of our hospital where they were treated for diabetes complications.

**Figure 1:**
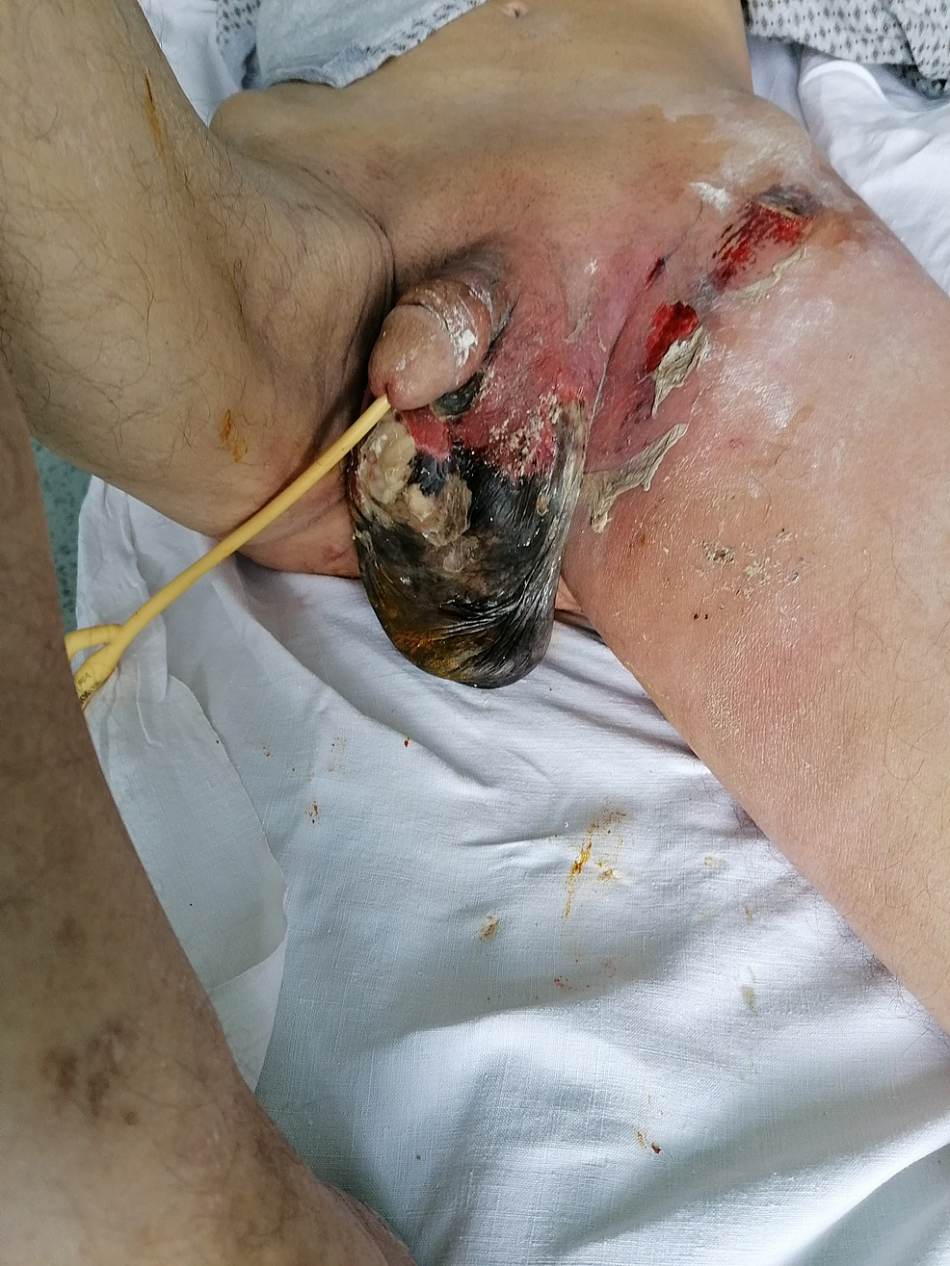
Patient with infected perineum and scrotum

**Figure 2:**
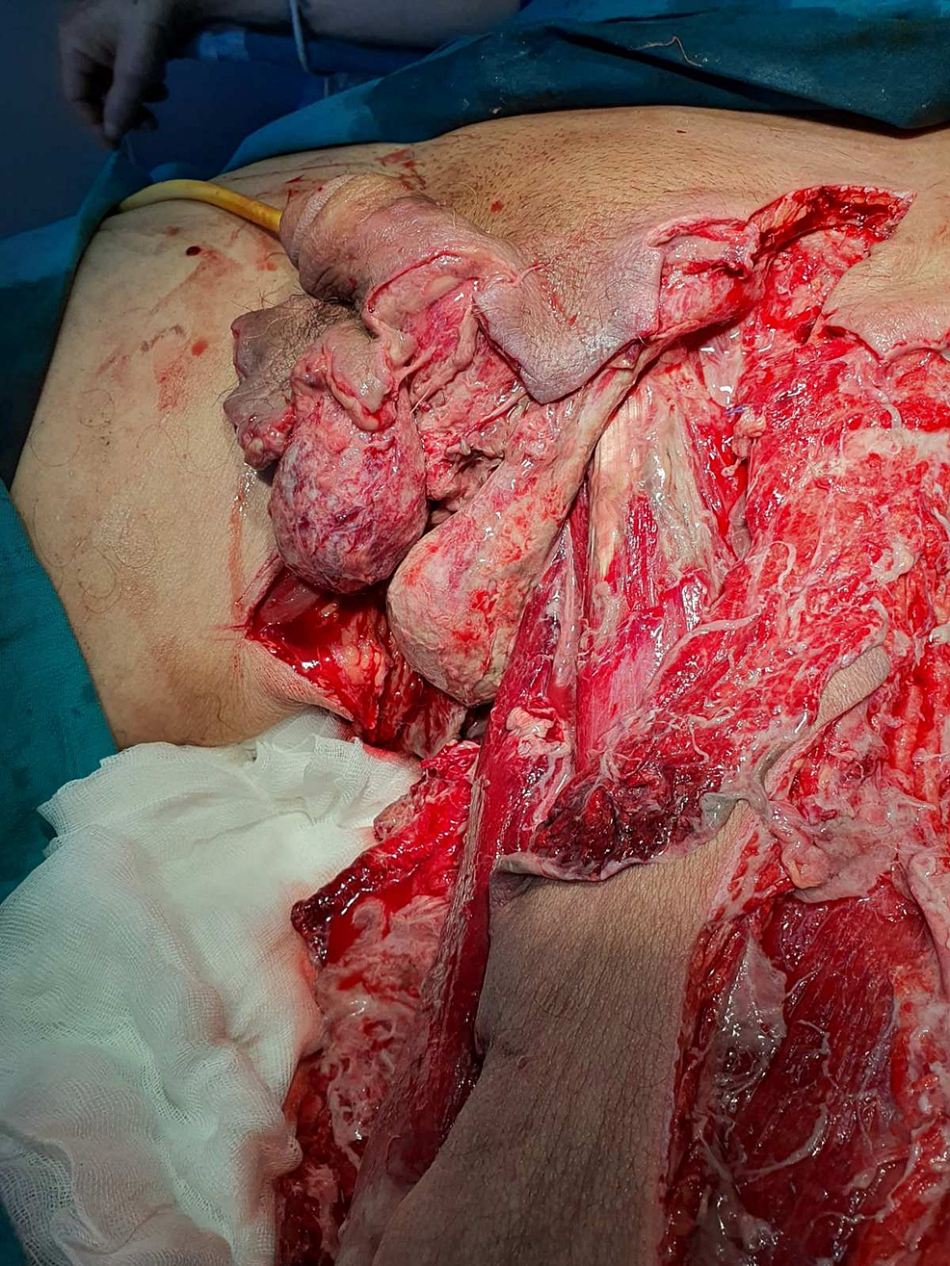
A: Intraoperative finding for patient No. 11: debridement of the left and right groin extending to the left thigh and scrotum

**Figure 3:**
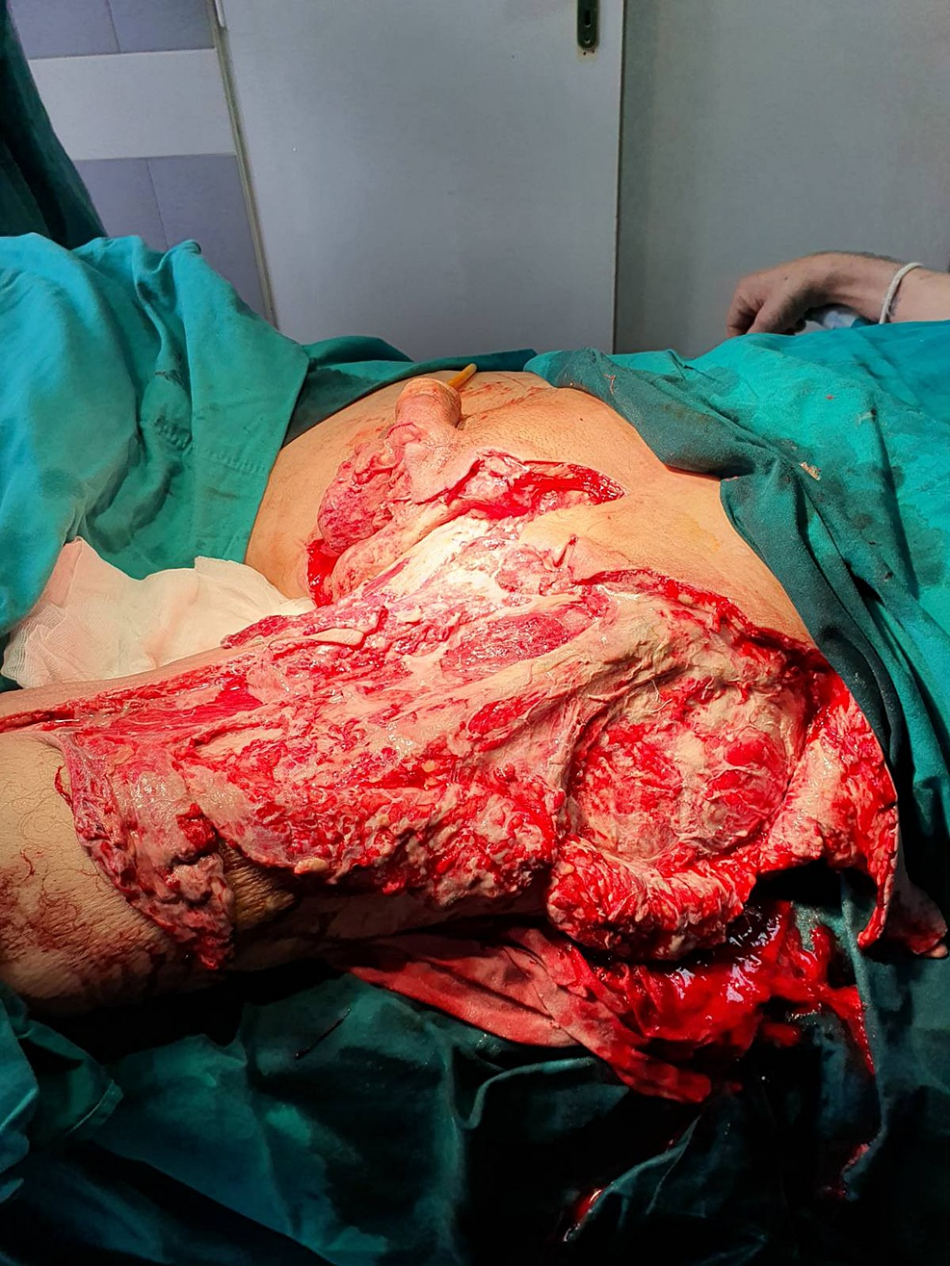
B: Intraoperative finding for patient No. 11: debridement of the left and right groin extending to the left thigh and scrotum

Fifty-three percent (53%) of patients were smokers, but none of them had a history of chronic alcohol consumption. All of them had at least one comorbidity and 61% had three or more. Diabetes, cardiovascular diseases, and obesity were the most common comorbidities (Table 1). Initial infected sites and areas of surgical debridement are presented in Table 2 and Figure 1. Only three (23%) patients had one surgical intervention; most had two or more. The mean number of surgical interventions to prevent the spread of NF was 2.3 (Table 2). Surgical procedures involved the excision and debridement of necrotic tissue to a macroscopically clear visible tissue (Figures 2-3). All patients received the first surgical debridement in the first 24h; the fastest was within 4h after admission to surgery. Sixty-one percent (61%) of the surgical procedures were done under general anesthesia and 39% under spinal anesthesia. All wounds were treated with local antiseptic dressings, two to three times per day, and left to heal per secundam.

**Table 1:** Patients' comorbidities DM: diabetes mellitus; HBP: high blood pressure; CKD: chronic renal disease; HCV: hepatitis C virus

Patients	DM	HBP	CKD	HCV	Obesity	Heart disease	Malignancy
1.	+	+	+		+	+	
2.	+	+			+		
3.	+	+			+		
4.	+	+				+	
5.	+	+			+	+	+
6.	+				+		
7.				+			
8.	+	+	+		+	+	+
9.		+			+		
10.	+	+			+	+	
11.	+	+				+	
12.		+					
13.		+					

**Table 2:** Initial infected site, surgical debridement region, and number of surgical debridement

	Initial infected site	Surgical debridement region	Number of surgical debridements
1.	Vulvar regions	anterior abdominal wall, perineum, inguinal region	4
2.	Umbilical hernia operation	anterior abdominal wall	6
3.	Perianal fistula	perineum and scrotum, as well as to both gluteal regions of the perianal region	3
4.	Right groin	right femoral and pubic region, infraumbilically bilateral anterior abdominal wall, pubic and right gluteal region	2
5.	The site of the operative incision after uterine tumor surgery	anterior abdominal wall	2
6.	Right femoral region	right femoral and inguinal region and perineum	1
7	Upper arm after IV drug use	left arm and anterior wall of left hemithorax	2
8	The site of the operative incision after uterine tumor surgery	anterior abdominal wall	2
9	Trauma	left thigh ( lateral and posterior region)	1
10	Perianal abscess	perineum, gluteal, and perianal region	2
11	Unknown	anterior abdominal wall, perineum, both inguinal region, scrotum, both femoral region	2
12	Surgical intervention	lower leg	1
13	Perianal abscess	perianal and gluteal region, perineum	2

Treatment of patients with NF requires a multidisciplinary approach by physicians of different specialties so consultations were done with an internist, endocrinologist, cardiologist, and nephrologist, due to the comorbidities during hospitalization. All patients received a combination of three antibiotics. Before surgery, they were usually treated empirically with antibiotics metronidazole, ceftriaxone, and gentamicin, and after obtaining microbiological results and antibiograms or after worsening of the local finding, combinations of the following antibiotics were used: imipenem, vancomycin, piperacillin/tazobactam, clindamycin, and ciprofloxacin. Patients' initial laboratory findings and Laboratory Risk Indicator for Necrotizing Fasciitis (LRINEC) score on admission are presented in Table 3. The median length of the hospitalization of these patients was 31 days.

**Table 3:** Initially laboratory findings and LRINEC score at admission WBC: white blood cells; RBC: red blood cells; BMI: body mass index; HGB: hemoglobin; LRINEC: Laboratory Risk Indicator for Necrotizing Fasciitis

Patients	M/F	Year	CRP	WBC	RBC	HGB	Glycemia	Creatinine	BMI	Na+	LRINEC
1.	F	53	308.7	36.3	5.02	152	5.2	76	31.8	136	6
2	F	59	296.5	39.2	4.07	134	25.4	88	40.2	139	8
3	M	54	403.7	26	4.08	121	41	222	37.5	135	11
4	F	64	350	27	4.34	129	32	158	26.7	135	10
5	F	60	253	21.4	3.56	112	16	148	35.8	136	7
6	F	40	498.6	33.5	4.62	122	25	120	51.9	137	8
7	M	27	289.6	21.5	3.24	113	9	98	20.1	141	6
8	F	73	367	28.3	3.01	101	19	121	34.5	139	9
9	M	59	271	19	5.09	145	16	91	30.9	140	5
10	F	54	221	23	4.43	131	19	167	32.1	136	9
11	M	61	351	32.7	2.95	101	39	189	27.2	134	12
12	M	64	169	21	3.88	121	15	100	26.1	139	5
13	F	54	201	19	4.76	127	18	99	27.7	136	6

All patients had swabs taken for microbiological analysis of the pathogen. The most common causes of infection were *Klebsiella spp, Pseudomonas aeruginosa, S. pyogenes, *and *S. aureus*.

Before hyperbaric oxygen therapy (HBOT), colon surgery was performed according to the Hartman procedure in one patient. Conservative treatment was continued with HBOT during the hospitalization. After they were discharged for outpatient treatment with a regular wound toilet and occasional surgeon's control, only two more patients had HBOT. One patient died during hospitalization while others were discharged for outpatient treatment.

## Discussion

Necrotizing fasciitis (NF) is a rare, life-threatening soft tissue infection that requires prompt surgical and medical treatment [7]. Many studies cite different data on the incidence of NF, and the difference is most often explained by the fact that this is a rare disease and that relatively small case series and cohorts are published. According to the annual incidence rate, ranging from 0.72 to 9.2 per 100,000 person-years [7-8], Bodansky et al. stated that, recently, there has been an increase in the incidence of NF in England as in other countries [4,9]. Overall, the mortality rate is high (median mortality 32.2%) [3,10] and ranges from 6% to 76% [11]. Arif et al. stated in their study that in the USA, 4.8 deaths per 1,000,000 people have been reported for years without a change in incidence between 2002 and 2013 [6]. In the study of Bodansky et al., the mortality rate was 16%. The authors of this paper did not have access to data on the frequency of NF in Serbia [12]. In our small series, one patient died (7.69%) by NF during hospitalization.

NF affects both sexes and all age groups but most of the published studies have revealed a predominance among the elderly, with a mean age of over 50 years. Also, they show that men are commonly affected with a male-to-female ratio of 3:1 [3,13], but some studies show different results with a male-to-female ratio up to 10:1 [14-15]. Older age does not directly affect survival [16-17]. Some studies report the occurrence of NF in middle-aged patients as well as in children [18]. Our results show that the mean age was 55 years and men represent 38% of all patients.

Although NF can occur at all ages, elderly patients have a poorer prognosis, often a more severe clinical picture, and a poorer disease outcome, mainly due to the increased incidence of comorbidities.

The dominant comorbid diseases in NF are diabetes mellitus (DM), hypertension, and obesity. In addition to them, significant comorbidities that affect the worsening of the prognosis are chronic renal failure, chronic heart disease, alcoholism, immunosuppression, systemic disorders, cirrhosis, local trauma, intravenous drug abuse, malnutrition, peripheral arterial disease [19-20]. In a study by Gonullu et al., more than half of the patients had at least one predisposing comorbidity and the most common (50%) comorbidity factor was diabetes mellitus [21]. Tarchouli et al. reported an incidence of diabetes mellitus in 38% and cardiovascular diseases (high blood pressure, heart disease) in 51%, with a significantly higher mortality rate in heart disease [22]. The effects of diabetes mellitus on mortality rate in NF is less clear, and several studies could not detect the correlation between mortality and DM [21]. In our study, DM occurs in 69%, obesity in 61%, high blood pressure in over 80%, heart disease in 46%, and chronic kidney disease (CKD) and malignancy in 15%. All patients had at least one comorbidity, and 61% had three or more.

Many microbiological epidemiology studies of NF have been reported trying to identify microorganisms as etiologic pathogens of NF. Most commonly, it is a polymicrobial infection caused by aerobic and anaerobic bacteria. Kim et al. reported that 73.9% had one or more identifiable pathogens [23]. Bair et al. stated that a single pathogen was found as the infectious agent in 60.4% and multiple pathogens in 19.8% in their study [24]. Studies show that one of the important pathogens in NF in the USA is *Staphylococcus aureus* [25], and methicillin-resistant Staphylococcus aureus (MRSA) was identified as a causative microorganism in 14.6% of complicated skin and soft tissue infections in 12 European countries [26]. The most isolated microorganisms in England were staphylococci and streptococci with an increase in Gram-negative species, predominantly *E. coli *and* Klebsiella pneumonia* [4,27].

Wound cultures were mostly monomicrobial in our patients, and the isolated microorganisms were *S. aureus, Klebsiella spp, *and* Pseudomonas aeruginosa* with almost the same frequency and a slightly higher percentage of *S. aureus*.

The treatment of NF requires early and aggressive surgical treatment, antibiotic therapy, and supportive care. Data from the literature shows that a delay of surgical treatment, as well as an insufficient surgical debridement, contribute to an increase in the mortality rate. Most patients are admitted in critical condition, often septic, so it is necessary to take intensive resuscitation measures. The most common places of changes on the skin, which are the entry points for the spread of infection are the perineum, anterior abdominal wall, scrotum, and extremities [28]. It is important to emphasize that often the changes on the skin are disproportionate to the size of the infection that spreads through the subcutaneous tissue. In addition to other conservative measures for the treatment of patients with NF, for successful treatment of NF, early and radical surgical intervention are essential. Better chances of survival have patients who have previously undergone surgical debridement with removal of necrotic and devitalized tissue. The mortality rate can be up to nine times higher when primary surgery is performed 24h after the onset of symptoms [28]. In patients with NF, reoperations are common. According to data in the literature, several dozen reoperations have been described in patients with complicated wounds, but most often they are performed from one to 10, on average two to three, times [29]. Our data matched the literature data.

Due to the severity and uncertainty of the disease, there was a need to develop a scoring system for weight assessment. The LRINEC proposed by Wong et al. is a scoring system designed to differentiate NF from other soft tissue infections [28]. Radiological diagnostic methods (X-ray, ultrasound, CT, and MRI scans ) have their place in the diagnosis and prognosis of NF and may be helpful, but since NF is spreading rapidly, they should certainly not affect the delay of definitive management [30].

As supportive therapy, HBOT can go a long way in treating infections such as NF but it is not crucial. Our data are in accordance with the literature.

## Conclusions

NF is a life-threatening disease with high mortality rates, which requires a multidisciplinary approach for treatment. Prompt recognition of necrotizing fasciitis is mandatory, and identifying risk factors as well as the understanding of NF will help physicians make the best clinical decisions when faced with this disease.
